# Tuberculosis without Tubercle

**Published:** 1910-01-22

**Authors:** 


### TUBERCULOSIS WITHOUT TUBERCLE.

In The Hospital for January 30 and May 1 of last year were summarised papers on morbid changes in the skin and elsewhere, claimed as atypical manifestations of tuberculosis. On the Continent this subject, with which Professor Landouzy's name is closely connected, is now receiving some considerable amount of attention, and a survey * of the whole question by Von Lautmann deserves, therefore, note. It will be useful first of all to recall shortly the history of the pathology of tuberculosis. To Laennec we owe the conception of nodular tubercle and of tuberculous infiltration. These, macroscopically and microscopically, were then studied by Addison and by Lebert, and, after Koch's great discovery had revealed an all-important factor in the problem, by Baumgarten. The histological account of the development of tuberculosis thus arrived at has hitherto hardly been questioned. For a disease to be classed as certainly tuberculous, the finding of a characteristic microscopical appearance in the tissue affected was deemed well nigh essential. This postulate is now beginning, in some quarters, to be questioned. In certain cases with no typical foci—no multiplication of fixed cells, no zone of leucocytes, no caseation, fibrillation or giant cells—two schools of French observers are now in the habit of making a confident diagnosis of tuberculosis.

Such cases may be found amongst those of hepatic disease. British physicians have noted a remarkable association of acute tuberculous pleurisy or peritonitis with apparently non-tuberculous cirrhosis of the liver. But, according to Gougerot, tubercle bacilli, without tubercles, have been found in ordinary cirrhotic liver substance, whilst ascitic fluid from these cases, injected into the peritoneal cavities of young dogs, will produce tuberculosis in 40 per cent. of instances. More startling than this, it is claimed that fatty and amyloid liver are both merely atypical modifications of hepatic tuberculosis, and that both may be experimentally reproduced in animals by injection of a virus containing tubercle bacilli. Besides these chronic forms of disease, Landouzy described (at the recent American Tuberculosis Congress) an acute general tuberculosis in which tubercle bacilli may be demonstrated in connection with the hepatic vessels without any associated tubercles. Next may be mentioned diseases of the skin. Erythema induratum and erythema nodosum, hitherto associated with rheumatism, are ranked as tuberculous on the strength of results reached in connection with the ophthalmo- and cuti-reaction, with their clinical course and their coincidence with other tuberculous affections. (See Marfan: *Press Médicale*, No. 51, 1909.) The same may be said of purpura; while similar points are held to establish the tuberculous character of lupus erythematosus, which may cause enlargement of regional glands, may occur round a tuberculous fistula, and may pass directly over into ordinary lupus. Keloid is another common affection claimed to be similarly out of its proper category. Gougerot and Laroche record experimental production of lichen scrofulosorum and of erythema nodosum. In the case of the skin, absence of typical tuberculous histological appearances is conjectured as being due to low virulence or numbers of the bacilli, or to the restraining influence of sunlight.

Besides the liver and skin, the nervous system may be the seat of atypical tuberculous manifestations. It has long been known that in tuberculous subjects a basal meningitis may occur in which a post-mortem search for tubercles is not rewarded with success. Morbid changes are limited to injection of the vessels of the pia mater, with a gelatiniform exudation in the subarachnoid space. The Lyons school of observers attribute even chorea, multiple sclerosis, various neuroses, and some cases of neuralgia to tuberculosis. What chiefly, however, associates the last-named school with the subject under notice is a similar opinion touching rheumatic arthritis. Sir Dyce Duckworth's observations on this particular point are too recent to need repetition here. But in spite of the many publications of Poncet and his followers, the profession in France is by no means convinced, and the question remains open.

The position, then, taken up by some leaders of French medicine is that although tubercle formation, naked eye and microscopic, is the most frequent manifestation of the disease we know under that name, yet it is not the only one; various diseases now wrongly regarded may be grouped together as polymorphic localisations of an infection with the toxin of the tubercle bacillus, rather than with the bacillus itself. Evidently the theory is one which may easily be pushed to extremes, but should improved methods of exact diagnosis show some truth in it, the fact would redound much to the credit of Professor Landouzy, who for the last 25 years has been turning thought in this direction.

* *Uber die erweiterte Tuberkulosediagnose* (*Bacillotuberculose non-folliculaire*). *Internationales Centralblatt f. d. gesamte Tuberkulose Forschung* III. No. 10. S. 435.

### Crystallisation of Bismuth Carbonate.

Mr. William Duncan, who has great experience in these matters, discusses why a precipitate occurs when the following apparently simple prescription is made up and kept for a while:—

℞ Liquoris bismuthi ... ... ... ... ℥j

Sodii bicarbonatis ... ... ... ... ʒij

Aquam ad ... ... ... ... . ... ℥vj

Misce: fiat mistura.

It was found that the medicine might remain clear under certain circumstances as long as three months, but then precipitated oxycarbonate of bismuth amorphously. A similar experiment was made, using the potassium instead of the sodium salt, and more of it, as in the following prescription:—

℞ Liquoris bismuthi ... ... ... ... ℥j

Potassii bicarbonatis ... ... ... ... ʒiv

Aquam ad ... ... ... ... ... ℥vj

Misce: fiat mistura.

A precipitate formed in time, and upon microscopical examination this was found to be composed of minute crystals. Analyses proved it to be a bismuth carbonate, not hitherto obtained in crystalline form.

